# Immunoreactivity to metal and silica associates with sarcoidosis in Dutch patients

**DOI:** 10.1186/s12931-020-01409-w

**Published:** 2020-06-08

**Authors:** E. Beijer, B. Meek, X. Bossuyt, S. Peters, R. C. H. Vermeulen, H. Kromhout, M. Veltkamp

**Affiliations:** 1 Interstitial Lung Diseases Center of Excellence, Department of Pulmonology, St. Antonius Hospital, Koekoekslaan 1, 3435 CM Nieuwegein, The Netherlands; 2 Department of Medical Microbiology and Immunology, St. Antonius Hospital, Nieuwegein, The Netherlands; 3 Laboratory Medicine, University Hospital Leuven, Leuven, Belgium; 4 Department of Microbiology and Immunology, KU Leuven, Leuven, Belgium; 5 Institute for Risk Assessment Sciences, Utrecht University, Utrecht, The Netherlands; 6 Department of Pulmonology, University Medical Center, Utrecht, The Netherlands

**Keywords:** Sarcoidosis, Aluminium, Beryllium, Zirconium, Metals, Silica

## Abstract

**Background:**

Involvement of metals or silica in the pathogenesis of sarcoidosis has been suggested by several case reports and specific epidemiological studies. However, the combination of occupational exposure and an immunological reaction has not been studied before in a group of sarcoidosis patients and non-sarcoidosis controls.

**Methods:**

In 256 sarcoidosis patients and 73 control patients with obstructive sleep apnea, exposure to metal and silica was assessed using a questionnaire consisting of a complete occupational history subsequently linked to job-exposure matrices. Next, immunoreactivity to aluminium, beryllium, zirconium and silica was determined in 33 sarcoidosis and 19 control patients using a lymphocyte proliferation test.

**Results:**

In sarcoidosis, 83 out 256 patients (32.4%) had occupational exposure to metals or silica, compared to 24.7% in the control group (*p* = 0.21). A significantly higher percentage of the sarcoidosis patients tested showed immunoreactivity to metals or silica compared to the control group (21.2 and 0% respectively, *p* = 0.039).

**Conclusions:**

Immunoreactivity to silica and metals was only found in sarcoidosis patients, supporting the hypothesis that these antigens may be involved in the pathogenesis of a distinct subgroup of sarcoidosis patients. This indicates that when searching for causative agents in sarcoidosis patients, besides beryllium, also zirconium, aluminium and silica deserve clinical investigation.

## Background

Sarcoidosis is a systemic disease characterized by the formation of noncaseating granuloma, mostly affecting the lungs, skin, eyes and lymph nodes [1]. The aetiology of sarcoidosis remains unclear to date and possible etiologic factors such as metals or silica are not included in the current work-up of sarcoidosis. When beryllium exposure is clinically suspected in patients with sarcoidosis, chronic beryllium disease (CBD) can be excluded by testing for beryllium immunoreactivity using a Lymphocyte Proliferation Test (LPT). This is important since CBD can be histopathological and clinical indistinguishable from sarcoidosis [2]. Also other antigens such as aluminium, zirconium and silica are suggested to play a role in the pathogenesis of sarcoidosis [3–7]. At present, however, a causal relationship between these antigens and sarcoidosis has not been established. First because studies using a control group are lacking and second because exposure is difficult to assess. Identification of possible etiologic antigens in sarcoidosis is important since it may lead to a more personalized treatment regime. Retrospective studies on CBD showed that termination of beryllium exposure could lead to disease reversal [8, 9]. Avoiding exposure might therefore be the first step in the treatment of sarcoidosis patients in whom metal or silica might be involved in the disease pathogenesis.

Although involvement of metals and silica in sarcoidosis pathogenesis has been suggested by several case reports and epidemiological studies, it has not been systematically studied in a larger group of sarcoidosis patients. The aim of this study was to characterize both occupational exposure and immunoreactivity to metals and silica in a group of sarcoidosis patients and controls. Since sarcoidosis is a very heterogeneous disease, we hypothesize that metals or silica are involved in the pathogenesis of the disease in only a subgroup of patients. Therefore, we have investigated whether occupational history linked to a job exposure matrix (JEM) can be an appropriate tool to identify which sarcoidosis patients have to be further tested for metal and silica immunoreactivity.

## Material and methods

### Study subjects

Sarcoidosis patients visiting the Interstitial Lung Disease (ILD) outpatient clinic of St. Antonius Hospital (Nieuwegein, The Netherlands) received an invitation letter to participate in the study. The diagnosis of sarcoidosis had been established according to the criteria of the American Thoracic Society/European Respiratory Society [10]. The characteristics of the sarcoidosis patients, including age at diagnosis and inclusion, Scadding stage at diagnosis and inclusion, organ involvement and the use of immunosuppressive medication, were collected from medical records. As a control group, patients with obstructive sleep apnea (OSA) visiting the Pulmonary outpatient clinic of the St. Antonius Hospital were asked to participate. Patients diagnosed with OSA were used as controls based on the fact that there is no relationship between environmental triggers and development of OSA [11]. This study was approved by the Medical research Ethics Committees United (MEC-U) of the St. Antonius Hospital (R14.023) and written informed consent was obtained from all participants.

### Assessment of metal and silica exposure

Sarcoidosis and control patients received an online questionnaire asking for a complete occupational history. The patients’ job histories were coded using the International Standard Classification of Occupations 1968 (ISCO-68) [12]. This classification system consists of seven major groups, which are subdivided into several minor groups. These minor groups are further divided into unit groups, and eventually into single jobs. Every single job has a unique 5-digit code. This allows jobs to be organized into clearly defined sets according to the tasks and duties undertaken in the job. The complete job history, until the diagnosis of sarcoidosis, was taken into account. Occupational exposure to silica, and the metals nickel and chromium was assigned through the DOM-JEM developed by experts in the Netherlands for application in general population studies [13]. Occupational exposure to metals in general was assigned through the ALOHA + JEM, developed by the same experts [14]. Because this JEM needs jobs coded into ISCO-88 [15], initial ISCO-68 job codes were recoded into ISCO-88 codes.

### Lymphocyte proliferation tests to determine metal and silica immunoreactivity

#### Patient selection

A random selection of 45 sarcoidosis patients with occupational exposure to silica or metals were asked to donate a blood sample for a LPT on beryllium, aluminium, zirconium and silica. From the group of sarcoidosis patients without exposure, 10 patients who had an appointment in the near future were asked for the LPT as well. Those patients were selected in such way that age and sex did not differ from the exposed sarcoidosis patients invited for the LPT. The first 20 control patients included were also asked to donate a blood sample.

#### Melisa®

Blood was drawn in citrate tubes and sent within 24 h to Prohealth Medical, Nederweert, The Netherlands, where the memory lymphocyte immunostimulation assay (MELISA®) LPT was performed. MELISA® is a clinically validated blood test that detects type-IV allergy (sensitization) to multiple metals [16, 17]. Furthermore, it can also be used to determine sensitization for silica [18]. Lymphocytes were isolated from citrated blood, and 1*10^6^ cells were incubated for 5 days in HEPES-buffered media containing at least two consecutive dilutions of an antigen: aluminium nitrate, beryllium sulphate, silicon dioxide or zirconium dioxide. After cultivating for 5 days, proliferation was measured by incorporation of H^3^-labelled thymidine. Radioactivity was measured with a scintillation counter (Microbeta, Perkin Elmer). Stimulation indexes (SI) were calculated by dividing the antigen specific counts per minute by the mean counts per minute of the basal controls. SI of ≥3.0 were considered as a positive test results, based on the MELISA® guidelines [16].

#### Beryllium LPT

Patients with an SI of ≥3.0 for beryllium were invited to donate a second blood sample, which was sent to the department of Laboratory Medicine, University Hospitals Leuven, Belgium, where a second beryllium LPT was performed according to the protocol described in the ATS official statement on diagnosis and management of beryllium sensitivity and chronic beryllium disease [19]. Briefly, 1.8*10^5^ PBMCs/200 μL were incubated for 6 days in medium (RPMI-1640 GlutaMax supplemented with 5% autologous plasma and penicillin/streptomycin) containing concentrations of 10^− 4^ M till 10^− 10^ M BeSO_4_. Cell proliferation was measured by incorporation of H^3^-labelled thymidine. An SI of ≥3.0 was considered positive. The highest SI measured for each sample was used in the analysis. For calculating the correlation between the SI for beryllium obtained by MELISA® and obtained in Leuven, one additional patient, who had already performed both test for clinical evaluation, was included.

### Statistical analysis

Study data were collected and managed using REDCap electronic data capture tools hosted at St. Antonius hospital. Data were analysed using IBM SPSS statistics version 24. An unpaired T-test was used to compare numerical data between the sarcoidosis and control group. Non-parametric tests were used for non-normally distributed data (Mann-Whitney U test). Categorical data were compared between the sarcoidosis and control group using the Chi-squared test. If expected cell frequencies were below 5, Fisher’s exact test was used for categorical data up to two categories. Odds ratios were estimated for sarcoidosis and occupational metal and silica exposure. Correlation between the MELISA® LPT and validated beryllium LPT was determined with Spearman’s rho correlation. *P*-values < 0.05 were considered significant.

## Results

### Patient characteristics

A total of 590 patients were invited to participate in the study, of whom 314 responded and were willing to fill out the questionnaire. Finally, 256 sarcoidosis patients completed the occupational history and were included in the study. Seventy-three patients with OSA were included to serve as a control group. Demographics of all sarcoidosis and control patients are presented in Table 1. No difference in age at inclusion, sex or ethnicity was observed between the sarcoidosis and control patients. Only smoking status was significantly different between the sarcoidosis and control patients, with a higher proportion of never smokers in the sarcoidosis group.

**Table 1:** Demographics of sarcoidosis and control patients

	Controls (%) (*n* = 73)	Sarcoidosis (%) (*n* = 256)	P*
Male / Female	64.4 / 35.6	63.7 / 36.3	0.91
Age at diagnosis (Y)	N/A	43.57 ± 10.63	–
Age at inclusion (Y)	55.42 ± 14.37	54.50 ± 10.83	0.61
Ethnicity (W/B/O)	93.2 / 2.7 / 4.1	93.4 / 3.5 / 3.1	0.88
Smoking status ^a^			
(Never/former or current)	34.2 / 65.8	57.7 / 42.3	< 0.001
Scadding stage at inclusion			
(0/I/II/III/IV/ unknown)	N/A	30.1 / 9.8 / 18.4 / 12.9 / 26.2 / 2.7	
Extra pulmonary involvement		46.5	

Age at diagnosis and age at inclusion are shown as means ± std. deviation. Scadding stages were determined by chest X-ray. If Scadding stages at the time of inclusion were not available, Scadding stage was determined from the last obtained chest X-ray before the inclusion date. 0 = Normal chest radiograph; I = Bilateral hilar lymphadenopathy (BHL); II = BHL with pulmonary infiltrates; III = pulmonary infiltrates without BHL; IV = fibrosis.

*B* Black, *O* Other, *W* White

^a^ Smoking status was not available from 8 sarcoidosis patients. *Statistical differences for sex, ethnicity and smoking status were accessed using Pearson Chi-Square, statistical differences for age were calculated using an unpaired T-test

### No differences in occupational history and exposure between sarcoidosis patients and controls

The distribution of sarcoidosis patients and controls over the major occupational groups is shown in Table 2.

**Table 2:** Percentage of patients within ISCO-68 major groups and in minor groups of major group 7/8/9

Major occupational group	Sarcoidosis (%) (*n* = 256)	Controls (%) (*n* = 73)
0/1. Professional, Technical and Related Workers	34.8	30.1
2. Administrative and Managerial Workers.	11.7	11.0
3. Clerical and Related Workers	23.0	27.4
4. Sales Workers	9.8	15.1
5. Service Workers	13.3	16.4
6. Agricultural, Animal Husbandry and Forestry Workers, Fishermen and Hunters	3.5	2.7
7/8/9. Production and Related Workers, Transport Operators and Labourers	41.4	42.5
Minor occupational groups of major group 7/8/9		
70. Production Supervisors and General Foremen	3.5	4.1
71. Miners, Quarrymen, Well Drillers and Related Workers	0.4	0.0
72. Metal Processers	2.7	0.0
73. Wood Preparation Workers and Paper Makers	0.8	0.0
77. Food and Beverage Processers	3.1	4.1
79. Tailors, Dressmakers, Sewers, Upholsterers and Related Workers	1.2	1.4
81. Cabinetmakers and Related Woodworkers	0.4	0
83. Blacksmiths, Toolmakers and Machine-Tool Operators	3.1	6.8
84. Machinery Fitters, Machine Assemblers and Precision Instrument Makers (except Electrical)	9.4	8.2
85. Electrical Fitters and Related Electrical and Electronics Workers	10.9	9.6
87. Plumbers, Welders, Sheet Metal and Structural Metal Preparers and Erectors	7.4	2.7
88. Jewellery and Precious Metal Workers	0.8	0.0
90. Rubber and Plastics Product Makers	0.4	0.0
91. Paper and Paperboard Products Makers	0.0	1.4
92. Printers and Related Workers	0.4	0.0
93. Painters	1.6	1.4
94. Production and Related Workers Not Elsewhere Classified	1.6	0.0
95. Bricklayers, Carpenters and Other Construction Workers	4.7	8.2
97. Material-Handling and Related Equipment Operators, Dockers and Freight Handlers	3.5	4.1
98. Transport Equipment Operators	6.3	8.2
99. Labourers Not Elsewhere Classified	0.8	1.4

Percentages of sarcoidosis patients and controls are based on all jobs performed during working life until the diagnosis of sarcoidosis was made (sarcoidosis group) or until the moment of study enrolment (control group). If participants had more than one job within the same group, only one was counted. Minor occupation groups without sarcoidosis patients and controls are not shown

Percentages of sarcoidosis patients and controls are based on all jobs performed during working life until the diagnosis of sarcoidosis was made (sarcoidosis group) or until the moment of study enrolment (control group). If participants had more than one job within the same group, only one was counted. Minor occupation groups without sarcoidosis patients and controls are not shown.

Almost all occupations classified by the JEMs as involving exposure to metals or silica, were jobs in major group 7/8/9 (Production and Related Workers, Transport Operators and Labourers). Hence we compared the distribution of sarcoidosis and controls patients also over the minor groups belonging to major group 7/8/9 (Table 2). No significant differences between sarcoidosis patients and controls were observed for any of these major or minor group. Occupational metal or silica exposure was assigned to 83 sarcoidosis patients (32.4%) and 18 control patients (24.7%) (*p* = 0.21) (OR = 1.47; 95% CI 0.81 to 2.97, supplementary Table 1). No significant differences were observed in occupational metal or silica exposure between the sarcoidosis and control group (Table 3).

**Table 3:** JEM assigned exposure of sarcoidosis patients and controls

JEM Exposure	Controls (*n* = 73)	Sarcoidosis (*n* = 256)	P*
Silica	4 (5.5)	19 (7.4)	0.57
Metals	17 (23.3)	75 (29.3)	0.31
Chromium	6 (8.2)	35 (13.7)	0.21
Nickel	6 (8.2)	31 (12.1)	0.35
Silica and/or metal	18 (24.7)	83 (32.4)	0.21

Values are shown as absolute numbers with percentages in brackets.

* Statistical differences were accessed using Pearson Chi-Square

### Immunoreactivity to metals and silica was only observed in sarcoidosis patients

Forty-five patients occupationally exposed to metals or silica were invited for the LPT, of whom 37 were willing to donate a blood sample. Unfortunately, LPTs on 11 samples could not be performed due to an insufficient number of lymphocytes. Ten sarcoidosis patients who had no exposure based on the JEMs were invited for the LPT as well. Of those 10 samples, 3 patients also had an insufficient number of lymphocytes. Of the 14 patients in whom the lymphocyte count was insufficient, 8 were on immunosuppressive medication and 6 were not. In total, a LPT was performed on samples of 33 sarcoidosis patients, of whom 26 had JEM assigned exposure and 7 had no JEM assigned exposure to either metals or silica. Of the 20 blood samples from control patients, 1 sample could not be analysed for technical reasons. Three of those 19 control patients had JEM assigned exposure to either metals or silica. No significant differences regarding ethnicity, age at time of the LPT, sex or smoking were observed between the sarcoidosis and control patients of whom an LPT was performed. We found that 45.5% of the sarcoidosis patients, compared to none of the controls, were on immunosuppressive drugs at the time of the LPT (*P* < 0.01) (Table 4).

**Table 4:** Demographics of the sarcoidosis and control patients who had an LPT performed

	Controls (%) (*n* = 19)	Sarcoidosis (%) (*n* = 33)	P*
Male / Female	68.4 / 31.6	90.9 / 9.1	0.06
Age at inclusion (Y)	60.53 ± 14.21	54.36 ± 11.18	0.09
Ethnicity (W/B/O)	94.7 / 0 / 5.3	97.0 / 3.0 / 0	0.31
Smoking status ^a^ (Never/Former or current)	47.4 / 52.6	62.5 / 37.5	0.29
Using immunosuppressive medication	0	45.5	< 0.01

Age at inclusion is shown as means ± std. deviation. *B* Black, *O* Other, *W* White

^a^ Smoking status was not available from 1 sarcoidosis patient *Statistical differences for sex was accessed using Fisher’s Exact Test, *P*-values of ethnicity, smoking status and use of medication were calculated using Pearson Chi-Square and statistical differences for age were calculated using an unpaired T-test

**Fig. 1:**
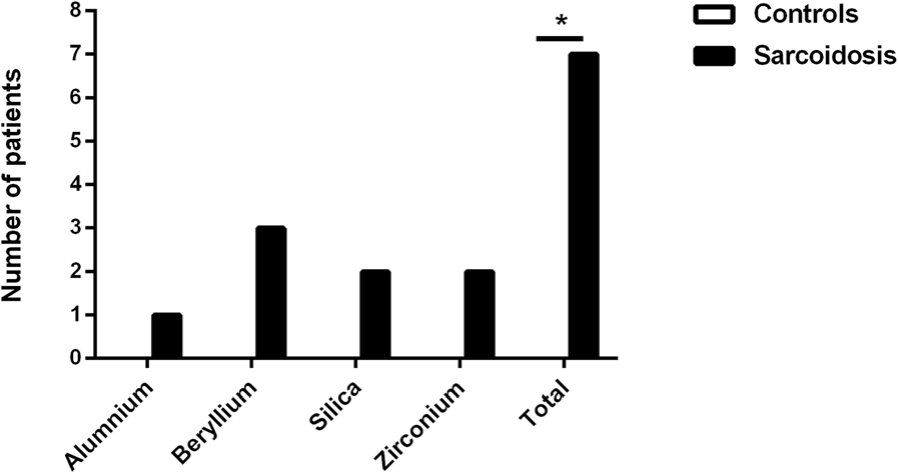
Number of sarcoidosis patients and controls with a positive LPT to the antigens tested. None of the control patients had a positive LPT for aluminium, silica, zirconium or beryllium. The difference between sarcoidosis patients and controls with a positive LPT to any of the antigens was significant (*P* = 0.039). A LPT was considered positive when the stimulation index was ≥3.0

Immunoreactivity was only observed within the sarcoidosis group. Seven (21.2%) sarcoidosis patients had a positive LPT for at least one of the antigens tested, compared to none of the controls (Fig. 1, *P* = 0.039). One sarcoidosis patient had a positive LPT for multiple antigens, namely zirconium and beryllium. In total, 3 sarcoidosis patients had an SI of ≥3.0 for beryllium and were invited for a second beryllium LPT using the ATS protocol. The highest SI values obtained by MELISA® for those three patients and the additional clinical patient were 3.96, 4.94, 9.05 and 24.48. The highest SI values of the second beryllium LPT following the ATS protocol for these patients were 1.27, 3.42, 5.93 and 379, respectively. When the results of those 4 patients were combined, a strong correlation of 1.00 (*P* < 0.01) was found between the beryllium SI of the MELISA® and LPT using the ATS protocol.

### Occupational exposure does not predict immunoreactivity

Occupational exposure as assigned by JEMs was compared with LPT results. Four of the 26 sarcoidosis patients (15.4%) with metal or silica exposure showed immunoreactivity to at least one of the antigens tested. Among the 7 sarcoidosis patients without JEM assigned exposure, 3 (42.9%) had a positive LPT for the antigens tested (Table 5). The difference in percentage positive LPTs between patients with and without JEM assigned exposure was not significant (*P* = 0.145).

**Table 5:** JEM assigned exposure of LPT positive sarcoidosis and control patients

Sarcoidosis or control	LPT positive for	SI	JEM assigned exposure
Sarcoidosis	Al	13.62	–
Sarcoidosis	Be	4.94	–
Sarcoidosis	Be, Zir	9.05, 3.51	–
Sarcoidosis	Be	3.96	Silica
Sarcoidosis	Si	3.20	Silica
Sarcoidosis	Si	6.90	Metals
Sarcoidosis	Zir	3.01	Metals

*Al* aluminium, *Be* Beryllium, *Si* Silica, *Zir* zirconium, *SI* Stimulation index

### Clinical phenotypes of LPT-positive sarcoidosis and LPT-negative sarcoidosis patients

Clinical characteristics of LPT-positive sarcoidosis patients and LPT-negative sarcoidosis patients are shown in Table 6. We also determined the clinical outcome status (COS), and divided it into three groups: resolved and minimal disease (COS 1,2,3,4), persistent disease without therapy (COS 5,6) and persistent disease with need for therapy (COS 7,8,9). No differences between the two groups were observed for any of the clinical characteristics or COS.

**Table 6:** Clinical features of LPT-positive and LPT-negative sarcoidosis patients

	LPT-negative (%) (*n* = 26)	LPT-positive (%) (*n* = 7)	P*
Male / Female	92.3 / 7.7	85.7/ 14.3	0.52
Age at diagnosis (Y)	43.46 ± 11.61	41.40 ± 7.97	0.66
Time after diagnosis (Y)	13.65 ± 12.39	9.21 ± 8.21	0.36
Medication	46.2	42.9	1.00
Scadding stage at diagnosis (0/I/II/III/IV, unknown)	11.5/26.9/42.3/3.8/0/ 15.4	0/42.9/28.6/0/14.3/ 14.3	0.36
Scadding stage at inclusion (0/I/II/III/IV, unknown)	38.5/7.7/11.5/11.5/30.8/ 0	28.6/14.3/14.3/0/28.6/ 14.3	0.42
Extra pulmonary involvement	57.7	57.1	1.00
COS	*n* = 21	*n* = 5	
Resolved & minimal disease	4.8	40	0.09
Persistent disease without therapy	33.3	20	1.00
Persistent disease with need for therapy	61.9	40	0.62

Age at diagnosis and time after diagnosis are shown as means ± std. deviation. *COS* Clinical outcome status. COS was defined 5 years after diagnosis. Information to determine COS was missing for 7 patients (5 LPT-negative and 2 LPT positive patients).

* Statistical differences for sex, medication, COS groups, extra pulmonary involvement, was accessed using Fisher’s Exact Test, *P*-values for scadding stages were calculated using Pearson Chi-Square. Statistical differences for age at diagnosis were calculated using an unpaired T-test and statistical differences for time after diagnosis was calculated using Mann-Whitney U test

## Discussion

This study is to our knowledge the first to combine both exposure analysis and lymphocyte proliferations tests for multiple inorganic antigens in patients with sarcoidosis and controls. We found immunoreactivity to metals and silica only in patients with sarcoidosis. Although a large group of patients with sarcoidosis was included, no differences in occupational exposure to metals and silica with the OSA control group was observed. Previous studies addressing such questions were either large epidemiological studies without immunological data, or smaller studies and case reports describing only one specific antigen [3, 7, 20, 21].

The fact that only patients within the sarcoidosis group demonstrated immunoreactivity for different antigens supports the concept of metals and silica involvement in the disease pathogenesis of a distinct subgroup of sarcoidosis patients. Different epidemiological studies have demonstrated a higher risk of sarcoidosis among persons occupationally exposed to silica [4, 22]. Immunoreactivity to silica in some sarcoidosis patients, suggests an underlying immunological mechanism that could explain these epidemiological observations. Regarding the role of zirconium and aluminium in sarcoidosis pathogenesis, no other studies than case-reports exist [3, 7, 23]. Müller-Quernheim and colleagues [20] established that in a well-defined cohort of biopsy-proven sarcoidosis patients, over 6% should be diagnosed with CBD based on the diagnostic criteria for this disease, which includes presence of non-caseating granulomas in lung or lymph node biopsies, beryllium exposure and sensitization [19]. In our cohort, 3 out of 256 sarcoidosis patients (1.2%) had a positive LPT for beryllium defined by MELISA®, of which two also had elevated SI values on the beryllium LPT using the ATS guideline protocol [19]. These results demonstrate that also in the Netherlands, patients with CBD can be identified within a group of biopsy proven sarcoidosis patients. This is a clinically relevant finding since termination of exposure to beryllium may be the first step in the treatment of CBD to prevent progression of the disease in these patients. In light of diagnostic criteria for CBD, it is tempting to speculate that patients with a positive LPT to zirconium, aluminium or silica, have a disease aetiology clinically comparable to that of CBD. In CBD, genetic susceptibility has been demonstrated, since a Glu at position 69 in the HLA-DPB1 gene is found in approximately 90% of patients compared to about 40% of healthy controls. The presence of this genetic variant has proven to be an important contributing factor to the development of CBD [24, 25]. Since immunoreactivity, but not occupational exposure, differed between patients and controls, genetic variants may very well be involved in the ability to respond to the metals and silica addressed in this study.

Another important conclusion from our study is that our screening method, including the questionnaire linked to the JEMs, appears unable to predict immunoreactivity. Almost half of our patients with a positive LPT were classified by JEMs as unexposed. The JEMs are designed to be very specific (i.e. high probability of exposure within a certain job) and will, by definition, not capture individuals experiencing coincidental high risk exposure while carrying out otherwise low-risk jobs [26]. It is important to state that even very low occasional exposures can be relevant, as suggested by the work of Infante et al. [27], who described that bystander beryllium exposure can lead to CBD. Perhaps a simple occupational history is not detailed enough, and additional (job-specific) questionnaires will be required to identify occasional/recreational exposures that can be sufficient to initiate sarcoidosis or a sarcoid-like disease. However, the addition of more detailed methods to establish exposure will also lead to less specificity, i.e. there will be a higher number of exposure assigned patients who may not show immunoreactivity. Furthermore, since even low non-occupational exposures [28] can be of relevance in light of immunoreactivity, it will be difficult to distinguish between meaningful and negligible exposures. Therefore, testing all sarcoidosis patients for immunoreactivity to metals and silica in future studies, irrespective of exposure, will probably be more convenient to address this issue.

A limitation of the study is that the group for whom a LPT has been performed was quite small. The LPT could not be performed on blood samples of some sarcoidosis patients, due to insufficient lymphocyte counts. Lymphocytopenia is a common observation in sarcoidosis patients [29], not related to immunosuppressive medication [30]. As a consequence, less LPTs were performed than anticipated. LPT subgroups proved to be too small to detect differences in clinical phenotype between the LPT-positive and LPT-negative sarcoidosis patients. Further studies are needed to indicate whether metal and silica immunoreactive patients have a distinct disease phenotype or particular genetic variants comparable to the Glu at position 69 in HLA-DPB1 in CBD. For beryllium, it has already been discovered that the Be^2+^ cation induces a conformational change in the HLA-DP2/peptide complex. Immunoreactivity develops when T lymphocytes regard the displaced peptide as foreign (non-self) and become activated [31]. Possibly, other metals, including aluminium and zirconium, may induce similar conformational changes in the MHCII-peptide binding complex. Therefore, future studies are warranted as well to clarify these underlying immunological mechanisms leading to aluminium, silica and zirconium immunoreactivity.

Another study limitation may be the sensitivity of the assays performed. For beryllium LPTs, about 1% false positive and 30% false negative results are observed. The specificity of this assay is quite high (0.969) although the sensitivity is determined to be lower (0.686) [32]. Data on sensitivity and specificity regarding aluminium, zirconium and silica LPTs are lacking but probably comparable. The MELISA® test is using a higher number of lymphocytes per test suggesting that the sensitivity could be slightly higher than the classical LPT [17]. Due to the low sensitivity observed for the beryllium LPT, it is possible that the number of sarcoidosis patients with metal/silica immunoreactivity we observed is an underestimation, and the real number of patients with a positive LPT may actually be higher.

In conclusion, immunoreactivity to silica and metals found in sarcoidosis patients only, supports the hypothesis that these antigens may be involved in the disease pathogenesis of a distinct subgroup of sarcoidosis patients. Since job history with assigned exposure did not differ between sarcoidosis patients and controls, our data indicate that even very low incidence or occasional exposures could be relevant in sarcoidosis patients. Future studies may consider testing all sarcoidosis patients for immunoreactivity without screening them on exposure beforehand. Finally, the data presented in this paper suggests that besides beryllium, also zirconium, aluminium and silica can be considered as possible antigens in this heterogeneous disease. Future studies should however be initiated to clarify underlying immunological pathways and mechanisms in order to establish a more definitive role for these antigens in sarcoidosis.

## Supplementary information

**Additional file 1 Table S1.** Estimated odds ratios for sarcoidosis and JEM assigned exposure

## Data Availability

The datasets used and/or analysed during the current study are available from the corresponding author on reasonable request.

## Abbreviations

BHL: Bilateral hilar lymphadenopathy
CBD: Chronic beryllium disease
COS: Clinical outcome status
ILD: Interstitial lung disease
ISCO-68: International Standard Classification of Occupations 1968
JEM: Job exposure matrix
LPT: Lymphocyte proliferation test
MEC-U: Medical research Ethics Committees United
MELISA: Memory lymphocyte immunostimulation assay
OSA: Obstructive sleep apnoea
SI: Stimulation index
