# Supplementary material for: Immunoreactivity to metal and silica associates with sarcoidosis in Dutch patients
Source: Respir Res. 2020 Jun 8;21:141. doi: 10.1186/s12931-020-01409-w (PMC7282065; doi:10.1186/s12931-020-01409-w)
Supplement: Supplementary file 1 — Additional file 1 Table S1. Estimated odds ratios for sarcoidosis and JEM assigned exposure [file 12931_2020_1409_MOESM1_ESM.docx]

**Supl. Table 1** Estimated odds ratios for sarcoidosis and JEM assigned exposure

| Exposure | Controls (%)  (n=73) | Sarcoidosis (%) (n=256) | OR (95% CI) | OR (95% CI)  Adjusted* |
| --- | --- | --- | --- | --- |
| Silica and/or metals | 24.7 | 32.4 | 1.47 (0.81, 2.65) | 1.62 (0.88, 2.97) |
| Silica | 5.5 | 7.4 | 1.38 (0.46, 4.20) | 1.52 (0.49, 4.70) |
| Metals | 23.3 | 29.3 | 1.37 (0.75, 2.50) | 1.48 (0.80, 2.76) |

.

*adjusted for smoking
